# Current situation and influencing factors of scientific literacy in fitness of Chinese children and adolescents

**DOI:** 10.3389/fpubh.2026.1760827

**Published:** 2026-02-11

**Authors:** Yanfeng Zhang, Deqiang Zhao, Xiaoxiao Chen, Jiaxing Chen, Chunmiao Wang, Sen Li, Hisashi Naito, Pengyu Deng

**Affiliations:** 1 National Physical Fitness Research Center, China Institute of Sport Science, Beijing, China; 2 Graduate School of Health and Sports Science, Juntendo University, Chiba, Japan; 3 School of Physical Education and Sports Rehabilitation, Jinzhou Medical University, Jinzhou, Liaoning, China; 4 Jiangsu Institute of Sports Science, Nanjing, China

**Keywords:** children and adolescents, influencing factors, scientific fitness literacy, self-determination theory, social–ecological theory

## Abstract

**Objective:**

This study aimed to develop and validate a scientific fitness literacy (SFL) assessment model for Chinese children and adolescents and to examine the associations between SFL and its key influencing factors within an integrated social–ecological and self-determination theory framework.

**Methods:**

Data were collected from 147,104 students (grades 4–12) across 31 provinces in mainland China using a multi-stage cluster random sampling design. SFL was assessed across four dimensions: knowledge, attitude, skills, and habits. The relative contributions of five influencing factors—lifestyle, individual socialization, family, school, and social environment—were analyzed using multiple regression models.

**Results:**

The overall SFL index was 65.8 (range 0–100), with knowledge (59.2) and skills (58.2) scoring below the passing threshold. Lifestyle (*β* = 0.264) and individual socialization (*β* = 0.240) were the strongest predictors of SFL, followed by family environment (*β* = 0.211), social environment (*β* = 0.153), and school environment (*β* = 0.101). The school environment exhibited the lowest marginal utility.

**Conclusion:**

The integrated framework provides a more comprehensive explanatory model for SFL than approaches that focus solely on internal factors. Interventions should prioritize lifestyle and socialization while placing greater emphasis on family and social environments rather than relying solely on school-based factors.

## 1 Introduction

Physical literacy (PL) was first proposed by British scholars in the early 21st century (1). It encompasses the motivation, confidence, physical competence, knowledge, and understanding that individuals develop for lifelong participation in physical activity (2). PL is recognized internationally and has influenced national sports and health strategies in various countries (3, 4). To ensure effective implementation, its conceptual domains are often adapted to local contexts (5). In China, policy discussions have explored integrating PL with indigenous cultural elements, such as Confucian and Taoist philosophies (6).

Understanding health behaviors often relies on two dominant theoretical frameworks: social–ecological model and self-determination theory (1, 7). The social–ecological model advocates for a multi-level analysis of behavior, extending beyond individual factors to include interpersonal, organizational, community, and policy-level environmental influences. However, critics note that this model sometimes lacks depth in explaining the internal psychological processes linking environmental inputs to behavioral outcomes. Conversely, self-determination theory provides a robust account of these internal mechanisms—specifically, how the satisfaction of basic psychological needs (autonomy, competence, and relatedness) fosters intrinsic motivation and sustained behavior. Yet, its primary focus on intra-individual processes may underrepresent the constraining or facilitating role of broader social and physical environments. Particularly in the context of Chinese children and adolescents, interventions based solely on self-determination theory might lack practical guidance for navigating complex environmental realities. Therefore, integrating these two perspectives offers a promising avenue for developing a more holistic explanatory framework for health behaviors. However, it is more accurate to describe this as constructing an integrative analytical framework rather than proposing an entirely new theory.

The health of Chinese youth underscores the urgency of this research. The 2014 National Physical Fitness Monitoring Bulletin revealed persistent challenges, including high rates of overweight and obesity and a general decline in physical fitness among children and adolescents (8). Research indicates that early scientific fitness literacy (SFL)—defined here as the knowledge, positive attitudes, practical skills, and sustainable habits necessary for children and adolescents to engage in safe, effective, and scientifically informed physical activity—is crucial for establishing lifelong healthy behaviors. Key factors influencing SFL include individual socialization (the process through which youth internalize fitness-related values, norms, and a sense of belonging through social interaction) and lifestyle (the degree to which regular, science-based physical activity is integrated into daily routines).

Building on the findings from a 2020 national survey, this study employs an integrative social–ecological and self-determination theory lens to identify and analyze factors affecting SFL among Chinese children and adolescents. We posit that when children’s needs for autonomy, competence, and relatedness in fitness contexts are supported by their environments, intrinsic motivation is more likely to develop, leading to greater integration of scientific fitness into their lives as a source of enjoyment and wellbeing.

Therefore, the primary aims of this study are: (1) to develop and validate an assessment model for SFL tailored to Chinese children and adolescents, and (2) to empirically examine the relative contributions of individual-level factors (e.g., lifestyle and socialization) and environmental-level factors (family, school, and community) to SFL within this integrated theoretical framework. This approach moves beyond traditional analyses focused solely on internal variables, offering a more comprehensive understanding of the determinants of SFL.

## 2 Participants and methods

### 2.1 Participants

The data for this cross-sectional study were derived from the Chinese National 2020 Survey on Sports and Fitness Activities of Children and Adolescents, commissioned by the General Administration of Sport of China. The target population was students in grades 4–12 (approximately ages 10–18) attending regular schools in China (31 provinces, autonomous regions, and municipalities, excluding Hong Kong, Macao, and Taiwan).

A multi-stage stratified cluster random sampling method was employed to ensure national representativeness. The sampling procedure consisted of four stages: (1) All 31 provincial-level regions were included. (2) Within each province, four prefecture-level cities were randomly selected, stratified by local Gross Domestic Product (GDP) to reflect economic diversity. (3) Within each selected city, one urban district and one rural county were randomly chosen. (4) Within each selected county/district, two primary schools, one junior high school, and one senior high school were randomly selected. Finally, 1–2 intact classes were randomly chosen from each sampled school, aiming for at least 60 valid respondents (approximately 30 boys and 30 girls) per class. This process yielded an initial sample from 1,112 schools.

Prior to data analysis, data cleaning was performed. Responses with extensive missing data on key variables (e.g., the entire Social–Emotional Learning scale left unanswered) were excluded. Obvious outliers and logically inconsistent answers were reviewed and treated as missing. The final valid sample comprised 147,104 participants. As shown in Table 1, the gender, urban–rural distribution, and grade composition of the final sample align well with the national demographic profile of this age group, demonstrating good representativeness. Since this study utilized a pre-existing national survey dataset designed for population-level estimation, no formal sample size calculation or power analysis was conducted *a priori*. The obtained sample size far exceeds the requirements for the planned multivariate analyses. To evaluate the statistical power of the available sample, a *post hoc* analysis was performed. For the core analytical models involved in this study (e.g., multilevel linear models, structural equation modeling), the current sample size provides statistical power exceeding 99% (*α* = 0.05) even for detecting very small effect sizes (e.g., Cohen’s d < 0.1 or ΔR^2^ < 0.01). This indicates that the sample is not only sufficient but also offers strong assurance for analyzing complex model structures and detecting subtle yet meaningful effects. Moreover, the large sample enhances the stability of parameter estimates and improves the reliability of subgroup analyses.

**Table 1:** Participants’ information.

Age (years)	Male (*n*)	Female (*n*)	Urban (*n*)	Rural (*n*)
10	8,120	8,248	8,337	8,248
11	8,232	8,229	8,249	8,229
12	8,272	8,223	8,243	8,143
13	8,210	8,214	8,219	8,114
14	8,143	8,092	8,243	8,092
15	8,125	8,178	8,225	8,078
16	8,143	8,132	8,211	8,032
17	8,113	8,145	8,213	7,945
18	8,175	8,110	8,274	8,009
Total	73,533	73,571	74,214	72,890

### 2.2 Physical literacy questionnaire

This study focuses on scientific fitness literacy (SFL), defined operationally as the integration of knowledge, positive attitudes, practical skills, and sustainable habits necessary for children and adolescents to engage in safe, effective, and scientifically informed physical activity. A self-report questionnaire was developed to assess SFL.

Questionnaire Development: The initial item pool and a four-dimensional framework (Knowledge, Attitude, Skills, and Habits) were generated based on a review of international PL literature and adapted to the Chinese context. Content validity was established through a two-round Delphi process with an expert panel (*n* = 8) consisting of senior scholars in physical education, public health, and child development. The panel rated the relevance, clarity, and importance of each proposed indicator. Subsequently, a pilot test was conducted with 320 students in Beijing and Tianjin. Exploratory factor analysis and item analysis (e.g., examining item–total correlations) were used to refine the scale, remove poorly performing items, and improve wording for clarity and age appropriateness across the targeted 10–18 age range. Measurement invariance across different age groups (e.g., children vs. adolescents) was not formally tested in this study, which is a noted limitation; however, the expert review and piloting aimed to ensure face validity and comprehensibility across the spectrum.

Final Questionnaire Structure: The final questionnaire contained two sections: (1) Sociodemographic information and (2) the SFL scale. The SFL scale consisted of four subscales: Knowledge: Assessed via true/false/unsure questions on basic fitness principles and safety. Attitude: Measured using a 5-point Likert scale (1 = Strongly Disagree to 5 = Strongly Agree) regarding the value and emotional inclination towards fitness. Skills: Combined self-assessment of competence and scenario-based questions on skills like workout planning and injury management. Habits: Assessed via self-reported frequency of fitness behaviors and active learning related to fitness.

Physical activity level was estimated within the Habits dimension by asking participants to recall the frequency, average duration, and perceived intensity (based on physiological cues: “stable breathing,” “increased breathing/light sweat,” and “rapid breathing/heavy sweating”) of moderate-to-vigorous physical activity in a typical week. We acknowledge the potential for recall bias and social desirability bias inherent in such self-reported measures.

Confirmatory factor analysis (CFA) using maximum likelihood estimation in Analysis of Moment Structures (AMOS) version 21.0 was conducted on the full sample to confirm the hypothesized four-factor structure of the SFL scale. The model showed acceptable fit to the data: χ^2^/df = 4.32, Comparative Fit Index (CFI) = 0.93, Tucker–Lewis Index (TLI) = 0.91, Root Mean Square Error of Approximation (RMSEA) = 0.06. Reliability was assessed using Cronbach’s alpha. The overall SFL scale demonstrated excellent internal consistency (*α* = 0.89). The subscales also showed good reliability: Knowledge (α = 0.78), Attitude (α = 0.85), Skills (α = 0.76), Habits (α = 0.82).

### 2.3 Theoretical framework and measures of influencing factors

**Figure 1:**
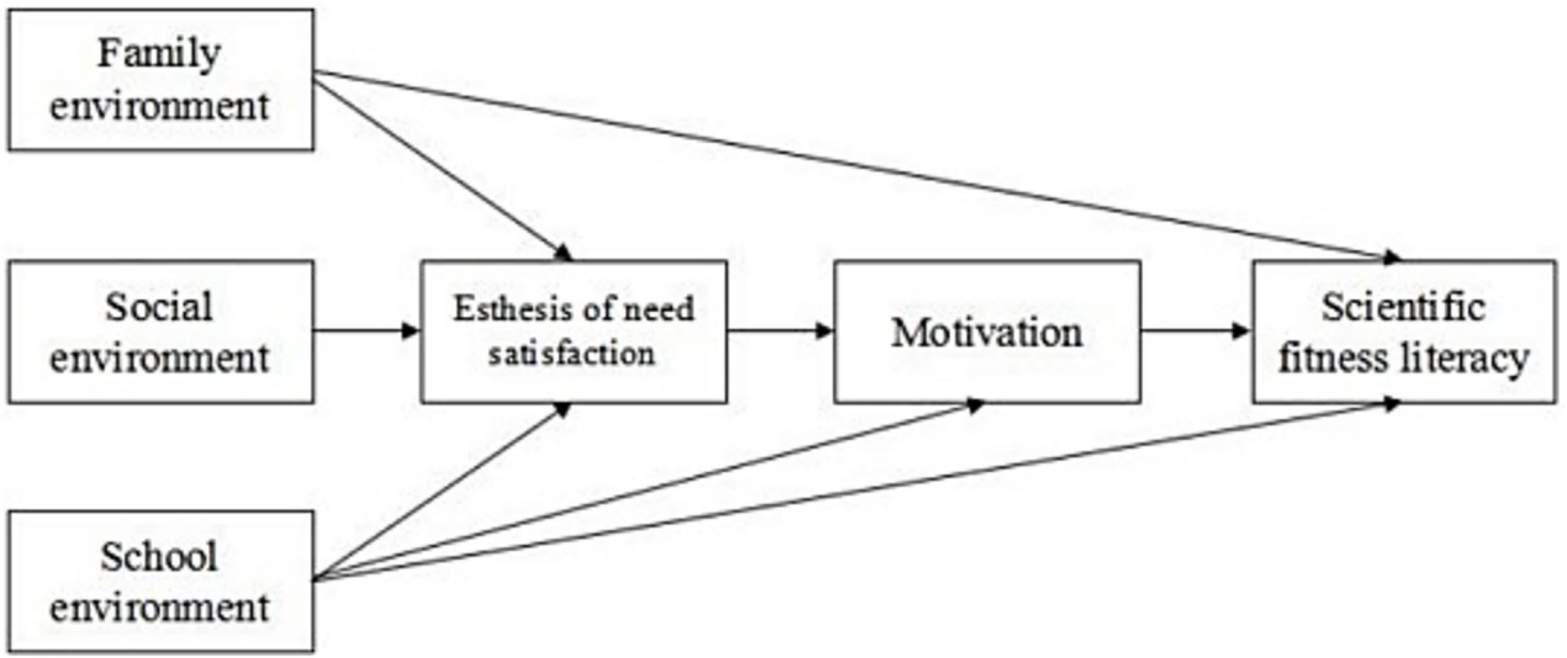
Integration of social ecology and self-determination theory.

The study is guided by an integrative framework (Figure 1) combining Social Ecology Theory and Self-Determination Theory (SDT). The framework posits that external environmental systems (Social Ecology) influence SFL primarily by facilitating or hindering the satisfaction of basic psychological needs (Autonomy, Competence, and Relatedness—from SDT), which, in turn, fosters high-quality motivation for sustained engagement in fitness activities.

#### 2.3.1 Operationalization of factors

Based on this framework, five sets of influencing factors were measured, corresponding to constructs from both theories: Lifestyle: An individual-level factor (aligned with SDT’s focus on behavioral integration) assessing the integration of physical activity into daily routines and leisure. Individual socialization: An individual/interpersonal-level factor reflecting the process of learning fitness norms, building relatedness through fitness, and internalizing fitness values (ties to SDT’s Relatedness and integrated regulation). Family environment: A micro-system environmental factor (Social Ecology) measured by parental attitudinal support, behavioral modeling (parental exercise), and financial support for fitness activities. School environment: A meso-system environmental factor (Social Ecology) measured by the implementation of physical education, school sports atmosphere, and availability of facilities. Social environment: A macro−/exo-system environmental factor (Social Ecology) measured by community fitness climate, accessibility of sports organizations, and perceived suitability of the natural environment for activity.

The scientific fitness literacy (SFL) evaluation system constructed in this study includes four first-level indicators: Cognition, Attitude, Behavior, and Habits, along with multiple second-level and third-level indicators (see Table 2).

**Table 2:** Evaluation index system of scientific fitness literacy.

Level indicators	The secondary indicators	Level-3 indicators
Scientific fitness awareness (25%)	Basic knowledge (70%)	/
	Basic concepts (30%)	/
Scientific attitude to fitness (25%)	Value judgment (30%)	/
	Emotions (30%)	/
	Behavioral tendency (40%)	/
Scientific fitness skills (25%)	Motor skills (50%)	Sports skills (50%)
		Safety protection skills (50%)
	Mental skills (50%)	Injury management skills (50%)
		Project selection skills (50%)
Scientific fitness behaviors and habits (25%)	Fitness study habits (50%)	Awareness of learning (50%)
		Learning style (50%)
	Fitness behavior habits (50%)	/

### 2.4 Statistical analysis

Confirmatory factor analysis (CFA) using maximum likelihood estimation in AMOS (version 21) was conducted to examine the factor structure of the SFL scale. Multiple linear regression models were built to assess the relative contribution of each influencing factor to the total SFL score. Models were built sequentially (Model 1–5) by adding factor groups to assess improvements in explanatory power (adjusted R^2^). Collinearity was assessed using tolerance and Variance Inflation Factor (VIF) statistics. ANOVA and *post hoc* LSD tests were used to compare mean SFL scores across different levels of each influencing factor. Statistical significance was set at *p* < 0.01.

## 3 Results

### 3.1 Analysis of SFL of children and adolescents

#### 3.1.1 Overall analysis of scientific SFL

**Figure 2:**
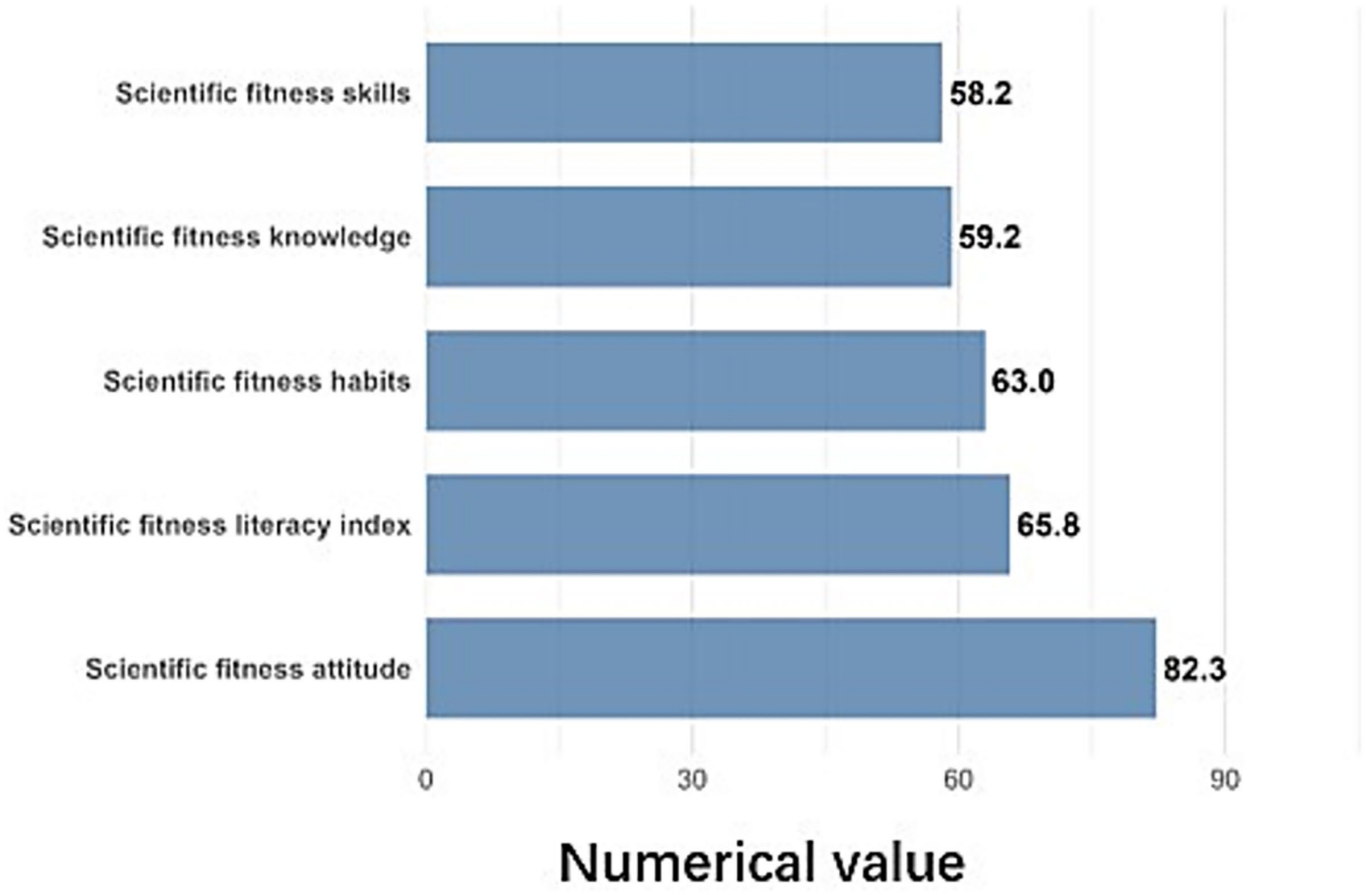
Scientific fitness literacy (SFL) index score.

In 2020, the overall scientific fitness literacy (SFL) index score for Chinese children and adolescents was 65.8 (on a scale of 0–100). The scores for the four dimensions were as follows: knowledge (59.2), attitude (82.3), skills (58.2), and habits (63.0) (see Figure 2). The overall SFL index indicates a medium-to-low level. While attitudes towards fitness were positive, knowledge and skills require further improvement, and scientific fitness habits need to be more fully developed. In terms of score distribution, 32.1% of participants scored between 60.0 and 69.9, 26.9% of them scored between 70.0 and 79.9, 10.7% of them scored between 80.0 and 89.9, and only 0.5% of them scored 90.0 or above.

#### 3.1.2 Analysis of scientific fitness knowledge

Children and adolescents demonstrated better grasp of basic fitness knowledge than of basic fitness concepts. The score for basic knowledge was 61.3, compared to 54.3 for basic concepts. Knowledge of pre-exercise warm-up activities was relatively high, with scores exceeding 87.0. Overall, 75.6% of participants could perform three or more warm-up activities skillfully. Notably, urban children and adolescents had a significantly higher scientific fitness knowledge index than their rural counterparts, with a mean difference of 2.8 points (*p* < 0.05).

#### 3.1.3 Analysis of scientific fitness attitude

Children and adolescents fully recognized the importance of fitness and held positive attitudes. The attitude survey covered three aspects: cognition, emotion, and behavioral tendency. Scores were high for cognition (89.6) and emotion (83.1), but lower for behavioral tendency (76.4), indicating that motivation to maintain fitness routines was somewhat insufficient. A high percentage strongly agreed that physical exercise is beneficial for enhancing fitness (86.2%), preventing excessive obesity (73.8%), and strengthening immunity (79.6%). Notably, positive attitudes towards scientific fitness declined with age: primary school students scored 85.0, junior high students 81.4, and senior high students 80.6.

#### 3.1.4 Analysis of scientific fitness skills

Overall, 53.7% of children and adolescents scored below 60 in scientific fitness skills. Skills were divided into motor skills and mental skills, with scores of 53.6 and 62.7, respectively. Motor skills related to the number of sports participated in and mastered were low (50.6 and 48.4). Mental skills, such as injury management and exercise planning, also scored low (49.5 and 40.6). These results indicate a need for improvement in both sport-specific training skills and safety competencies.

#### 3.1.5 Analysis of scientific fitness habits

The development of fitness learning habits was more advanced than that of fitness behavioral habits. The score for fitness study habits was 73.4, compared to 52.4 for fitness behavioral habits. Children and adolescents showed strong active learning awareness, with scores of 73.0 for learning fitness knowledge and 73.7 for learning fitness skills and methods. However, learning approaches remained relatively unitary and predominantly school-based. The score for the duration of physical exercise habit was only 49.0. Regionally, children and adolescents in North China had a significantly higher scientific fitness habits score (65.2) than those in other regions.

### 3.2 Analysis of factors influencing SFL in children and adolescents

#### 3.2.1 Analysis of variance for different factors

To examine the integrative framework combining Social Ecology and self-determination theory, we constructed multiple regression models by sequentially adding groups of influencing factors. The dependent variable in each model was the total SFL score. The models were as follows:

Model 1: Lifestyle.
Model 2: Lifestyle and individual socialization.
Model 3: Lifestyle, individual socialization, and family environment.
Model 4: Lifestyle, individual socialization, family environment, and school environment.
Model 5: Lifestyle, individual socialization, family environment, school environment, and social environment.

Table 3 summarizes the regression model results.

**Table 3:** Information table of multiple regression equation.

Independent variable	Adjusted R-square	Value of adjusted R-square	Standardized beta coefficient	*t*-test	*F*-test
Model 1	0.233	0.233			42,443.092**
Lifestyle			0.483	203.017**	
Model 2	0.342	0.109			35,527.302**
Lifestyle			0.38	165.176**	
			0.344	149.376**	
Model 3	0.41	0.068			31,260.581**
Lifestyle			0.277	117.794**	
Individual socialization			0.286	127.461**	
Family environment			0.292	123.967**	
Model 4	0.426	0.016			23,834.063**
Lifestyle			0.261	108.950**	
Individual socialization			0.259	112.697**	
Family environment			0.243	97.278**	
School environment			0.147	61.146**	
Model 5	0.446	0.020			19,900.537**
Lifestyle			0.264	110.121**	
Individual socialization			0.24	103.683**	
Family environment			0.211	82.465**	
School environment			0.101	40.189**	
Social environment			0.153	63.256**	

** Significance at the 0.01 level.

The results of the regression analysis are summarized in Table 3. The stepwise inclusion of factor groups led to consistent increases in the adjusted *R*^2^ values, supporting the explanatory power of the integrated framework. All models were statistically significant (*F*-test *p* < 0.01), and all independent variables made significant contributions (*t*-test *p* < 0.01).

In the final model (Model 5), the standardized beta coefficients (*β*) indicated the relative contribution of each factor, ranked from largest to smallest: Lifestyle (*β* = 0.264), Individual socialization (*β* = 0.240), Family environment (*β* = 0.211), Social environment (*β* = 0.153), and School environment (*β* = 0.101). This suggests that individual-level factors (lifestyle and socialization) were the strongest predictors, while the school environment had the lowest marginal utility in improving SFL.

#### 3.2.2 Collinearity test of all influencing factors of SFL

Collinearity diagnostics were performed for all variables in Model 5 (Table 4). Tolerance values were all close to 1 (range: 0.682–0.835), and Variance Inflation Factor (VIF) values were all below 2 (range: 1.197–1.466), indicating no substantial multicollinearity among the predictors. Eigenvalues were not close to zero, and condition indices were all below 30, with only the social environment factor having a condition index slightly above 15 (16.020). Given that other diagnostic metrics for this factor were within acceptable limits, significant collinearity was deemed unlikely. These results support the stability and clarity of the regression estimates.

**Table 4:** Collinearity test of all influencing factors of scientific fitness literacy.

Dimension	Tolerance	VIF	Characteristic value	Condition index
Lifestyle	0.779	1.284	0.327	4.111
Individual socialization	0.835	1.197	0.057	9.802
Family environment	0.682	1.466	0.041	11.631
School environment	0.715	1.399	0.033	12.850
Social environment	0.767	1.303	0.022	16.020

VIF, variance inflation factor.

### 3.3 The mean value analysis of the influencing factors of SFL in children and adolescents

**Figure 3:**
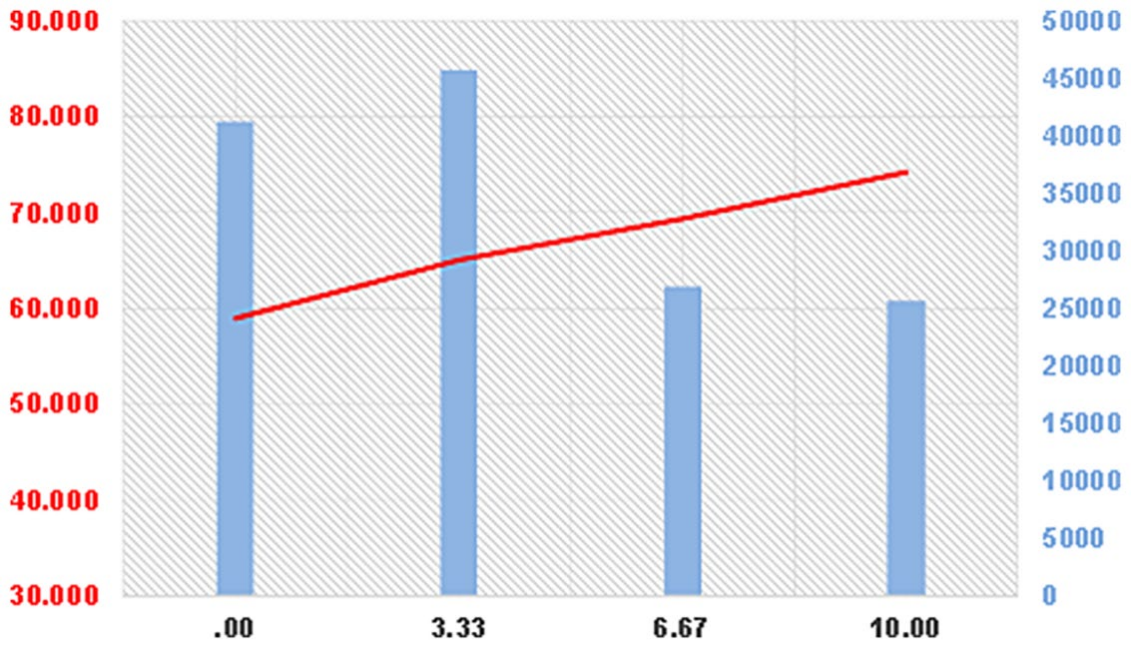
The mean distribution diagram of scientific fitness literacy among lifestyle groups. The red line values are shown on the left vertical axis, while the blue histogram values are on the right vertical axis. The values on the horizontal axis refer to the specific lifestyle score groupings; the blue histograms show the frequencies (sample sizes) corresponding to each lifestyle value, and those on the red line are the average values of the scientific fitness habit scores corresponding to the lifestyle value. For example, the lifestyle score of 6.67 (which is divided into four groups based on lifestyle scores) had a sample size of 26,943 and a corresponding average SFL score of 69.418 (the mean value for this group). Similarly, the mean distribution diagram of the other influencing factors among the groups is shown in Figure 4.

ANOVA and *post hoc* LSD tests were conducted to compare mean SFL scores across different levels (groupings) of each influencing factor. The mean distribution of SFL scores across lifestyle groups is illustrated in Figure 3.

The frequency distribution of the influencing factors revealed distinct patterns: Lifestyle scores were concentrated in the lower range (0.00–3.33 points), accounting for over 62% of the sample, suggesting room for better integration of fitness into daily life. Individual socialization scores were mainly concentrated at higher levels (8 points or above), accounting for 71%, indicating that most youth actively internalize fitness values through social interaction. Family environment scores followed an approximately normal distribution. School environment scores were skewed towards higher values, with 70% scoring 6 points or above, reflecting the structured nature of school-based physical activity.

**Figure 4:**
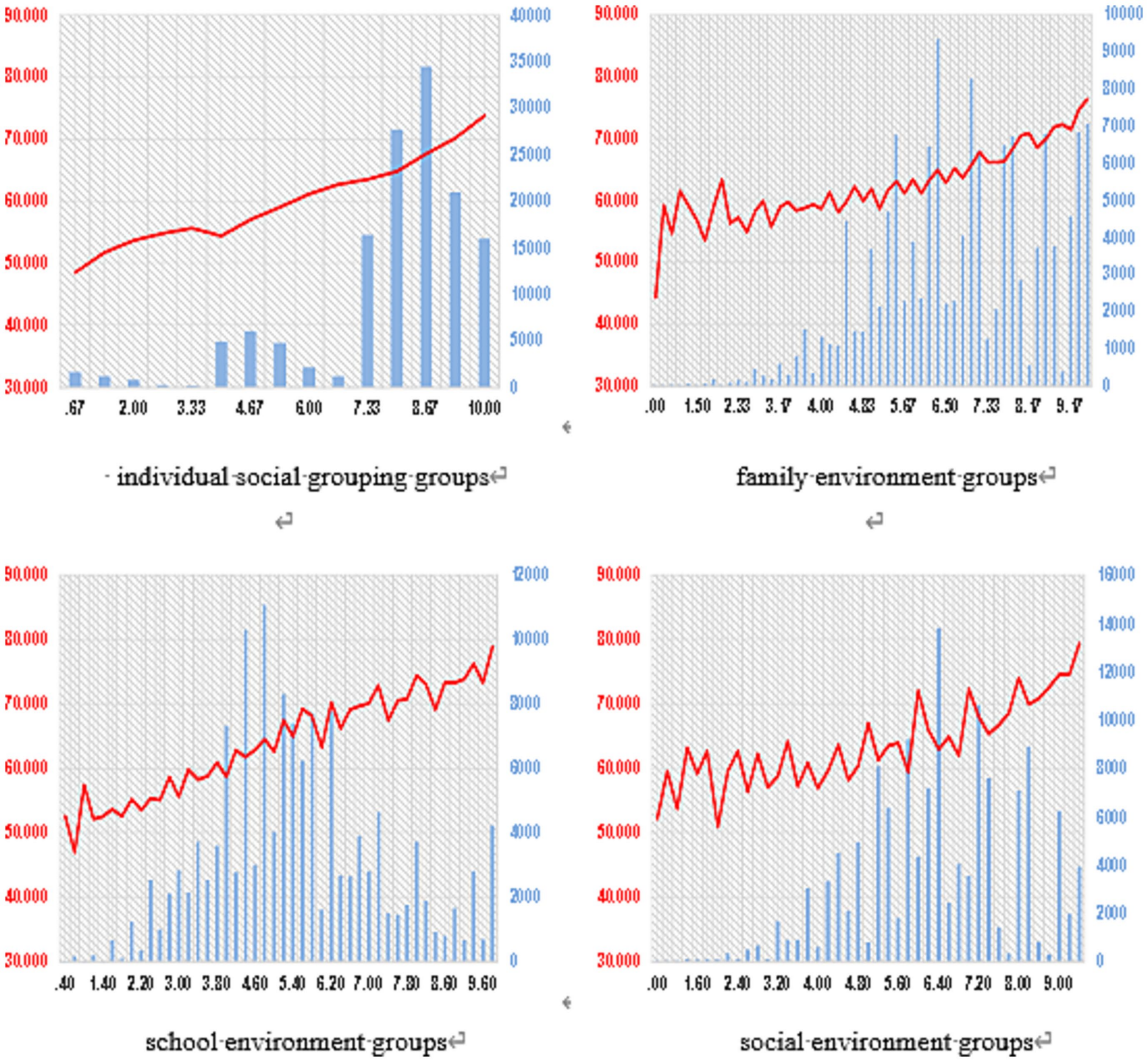
Mean distribution of scientific fitness behaviors among various factor groups.

The mean SFL scores (represented by red trend lines in Figure 4) for groups within each influencing factor generally showed a smooth, increasing trend as the group values increased, particularly for lifestyle and individual socialization. However, for family, school, and social environments, the trend lines exhibited more pronounced “sawtooth” or serrated patterns, indicating less consistent and more uncertain relationships between these environmental factors and SFL scores.

*Post hoc* LSD pairwise comparisons (detailed in Tables 5–9) showed that, at the 0.01 significance level, the number of non-significant differences between adjacent groups increased for the environmental factors (family, school, and social) compared to individual factors (lifestyle and socialization). This suggests that lifestyle and individual socialization exert a more direct influence on SFL, with a clearer corresponding relationship between these factors and SFL levels. In contrast, the impact of external environmental factors is more indirect and uncertain, with this uncertainty increasing as the environment becomes more distal (from family to school to community).

**Table 5:** Paired comparison between lifestyle groups.

I (lifestyle score)	J (lifestyle score)	Mean difference (I − J)	Standard error	Significance	95% Confidence interval difference	
					Inferior limit	Upper limit
0.00	3.33	−6.115	0.067	0.000	−6.246	−5.984
	6.67	−10.456	0.077	0.000	−10.607	−10.305
	10.00	−15.298	0.078	0.000	−15.451	−15.145
3.33	0.00	6.115	0.067	0.000	5.984	6.246
	6.67	−4.340	0.076	0.000	−4.488	−4.192
	10.00	−9.183	0.077	0.000	−9.333	−9.032
6.67	0.00	10.456	0.077	0.000	10.305	10.607
	3.33	4.340	0.076	0.000	4.192	4.488
	10.00	−4.842	0.086	0.000	−5.010	−4.674
10.00	0.00	15.298	0.078	0.000	15.145	15.451
	3.33	9.183	0.048	0.000	9.032	9.333
	6.67	4.842	0.086	0.000	4.674	5.010

Annotation: Dependent variable: Scientific fitness literacy score.

**Table 6:** Paired comparison between individual socialization groups (truncated).

I (Individual socialization score)	J (Individual socialization score)	Mean difference (I − J)	Standard error	Significance	95% Confidence interval difference	
					Inferior limit	Upper limit
3.33	0.67	7.142	0.855	0.000	5.467	8.817
	1.33	3.924	0.867	0.000	2.224	5.624
	2.00	2.029	0.893	0.023	0.079	3.780
	2.67	0.900	1.017	0.376	−1.094	2.894
	4.00	1.233	0.830	0.137	−0.394	2.860
	4.67	−1.432	0.828	0.084	−3.055	0.190
	5.33	−3.440	0.831	0.000	−5.068	−1.812
	6.00	−5.533	0.847	0.000	−7.192	−3.874
	6.67	−7.042	0.867	0.000	−8.742	−5.342
	7.33	−7.845	0.822	0.000	−9.455	−6.234
	8.00	−9.171	0.820	0.000	−10.779	−7.564
	8.67	−11.958	0.820	0.000	−13.565	−10.351
	9.33	−14.417	0.821	0.000	−16.027	−12.808
	10.00	−18.075	0.822	0.000	−19.686	−16.464

Annotation: Dependent variable: Scientific fitness literacy score.

**Table 7:** Paired comparison between home environment groups (truncated).

I (Family environment score)	J (Family environment score)	Mean difference (I − J)	Standard error	Significance	95% Confidence interval difference	
					Inferior limit	Upper limit
2.00	0.40	2.722	4.810	0.571	−6.705	12.149
	0.80	8.276	0.762	0.000	6.782	9.770
	1.00	3.108	0.714	0.000	1.709	4.506
	1.20	2.726	1.746	0.118	−0.697	6.149
	1.40	1.562	0.457	0.001	0.666	2.458
	1.60	2.687	0.955	0.005	0.815	4.560
	1.80	1.707	0.579	0.003	0.572	2.842
	2.20	−0.081	0.332	0.809	−0.732	0.571
	2.40	0.233	0.408	0.568	−0.567	1.032
	2.60	−3.395	0.343	0.000	−4.068	−2.722
	2.80	−0.349	0.326	0.285	−0.988	−291.000
	3.00	−4.596	0.342	0.000	−5.267	−3.925
	3.20	−3.058	0.314	0.000	−3.674	−2.442
	3.40	−3.614	0.332	0.000	−4.265	−2.962
	3.60	−5.673	0.316	0.000	−6.292	−5.054
	3.80	−3.399	0.295	0.000	−3.976	−2.822
	4.00	−7.543	0.328	0.000	−8.184	−6.901
	4.20	−6.429	0.288	0.000	−6.994	−5.864
	4.40	−7.541	0.324	0.000	−8.176	−6.906
	4.60	−9.245	0.287	0.000	−9.807	−8.682
	4.80	−7.390	0.311	0.000	−8.001	−6.780
	5.00	−1.087	0.041	0.000	−1.176	−1.014

Annotation: Dependent variable: Scientific fitness literacy score.

**Table 8:** Paired comparison between school environment groups (cut-off).

I (School environment score)	J (school environment score)	Mean difference (I − J)	Standard error	Significance	95% Confidence interval difference	
					Inferior limit	Upper limit
5.00	0.00	17.585	2.377	0.000	12.926	22.243
	5.00	2.671	5.032	0.596	−7.192	12.534
	0.83	7.349	1.461	0.000	4.485	10.214
	1.00	0.332	2.377	0.889	−4.326	4.991
	1.33	2.791	1.343	0.038	0.160	5.423
	1.50	5.107	2.694	0.058	−0.173	10.386
	1.67	8.443	1.288	0.000	5.918	10.968
	1.83	2.847	0.772	0.000	1.334	4.360
	2.00	−1.424	2.151	0.508	−5.640	2.792
	2.17	5.666	1.097	0.000	3.515	7.817
	2.83	1.987	0.629	0.002	0.754	3.219
	3.00	6.197	0.780	0.000	4.667	7.726
	3.17	3.028	0.446	0.000	2.153	3.903
	4.17	0.533	0.343	0.120	−0.139	1.204
	4.67	−0.424	0.310	0.172	−1.032	0.184
	4.83	1.979	0.312	0.000	1.368	2.590
	5.17	3.293	0.274	0.000	2.755	3.830
	5.33	0.262	0.221	0.237	−0.172	0.696

Annotation: Dependent variable: Scientific fitness literacy score.

**Table 9:** Paired comparison between social environment groups (truncated).

I (school environment score)	J (school environment score)	Mean difference (I − J)	Standard error	Significance	95% Confidence interval difference	
					Inferior limit	Upper limit
3.00	0.00	4.927	2.877	0.087	−0.711	10.566
	0.60	−2.442	2.015	0.225	−6.391	1.507
	1.00	3.431	4.118	0.405	−4.640	11.503
	1.20	−2.087	1.446	0.149	−4.921	0.747
	1.80	−5.599	1.056	0.020	−4.517	−0.379
	2.40	0.769	1.015	0.449	−1.220	2.758
	2.80	−1.637	0.944	0.083	−3.487	0.213
	3.40	−0.600	0.970	0.000	−8.971	−5.170
	3.60	−0.026	0.969	0.978	−1.926	1.873
	3.80	0.139	0.998	0.889	−1.817	2.095
	4.20	−6.604	0.929	0.005	−4.443	−0.801
	4.40	−1.600	0.925	0.000	−8.417	−4.791
	4.60	−1.023	0.938	0.276	−2.861	0.816
	4.80	−3.270	0.924	0.000	−5.080	−1.459
	5.00	−9.957	0.919	0.012	−4.117	−0.515
	6.00	−15.003	0.975	0.000	−15.637	−11.817
	8.60	−15.494	1.069	0.000	−17.589	−13.400
	9.00	−17.486	0.922	0.000	−19.293	−15.680
	9.20	−17.525	0.940	0.000	−19.366	−15.683
	10.00	−22.259	0.927	0.000	−24.075	−20.443

Annotation: Dependent variable: Scientific fitness literacy score.

### 3.4 The analysis of the influencing factors of SFL in children and adolescents

Multiple linear regression was conducted on the detailed items within each influencing factor to obtain standardized partial regression coefficients, indicating the relative contribution of each specific component.

#### 3.4.1 Way of life

**Figure 5:**
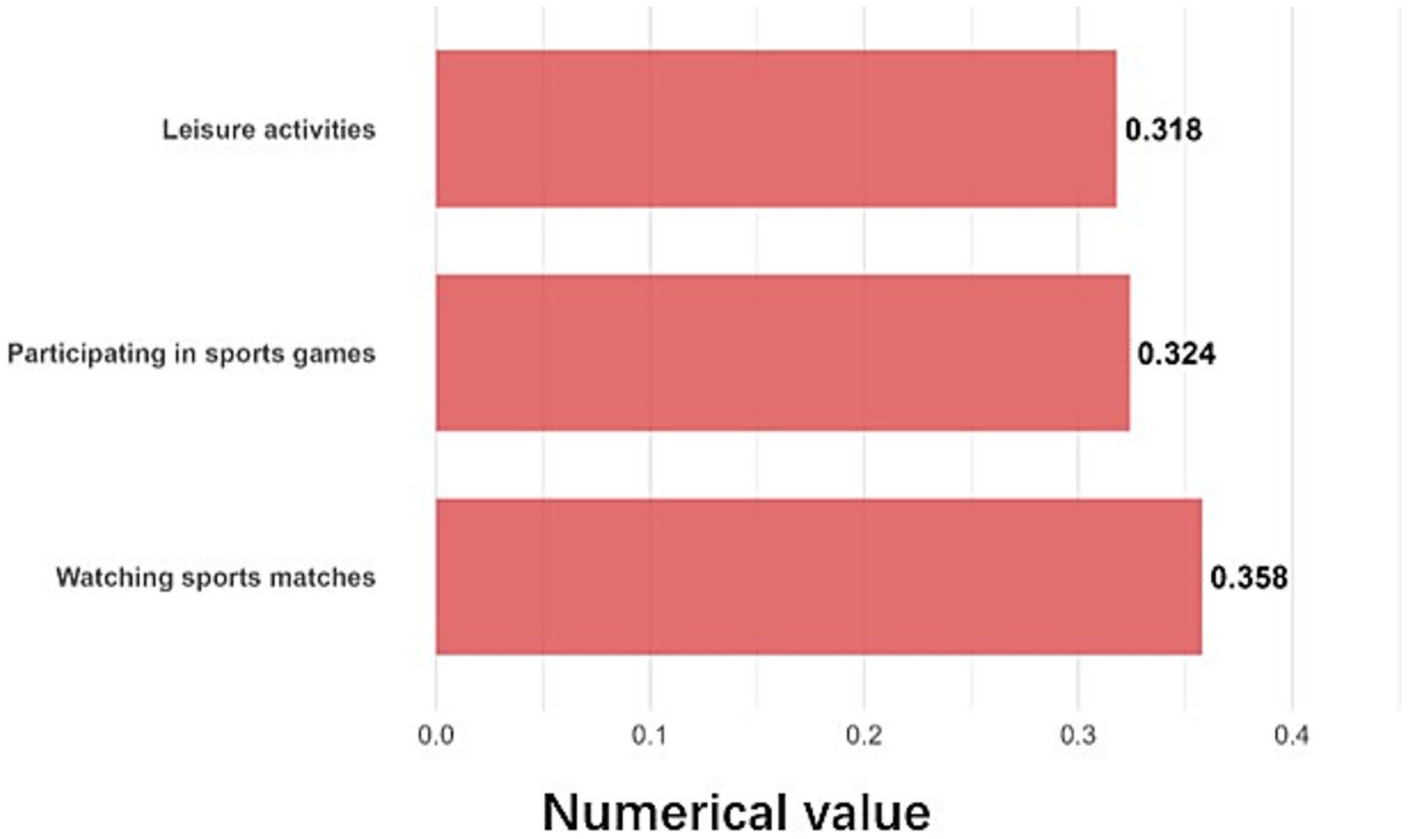
Details of the contribution of the lifestyle.

Lifestyle influenced SFL mainly through interpersonal and leisure-related components (Figure 5). The most significant items were: watching sports (*β* = 0.358), participating in sports (*β* = 0.324), and leisure activities after school and during holidays (*β* = 0.318). Children who watched sports had an average SFL index of 69.1, compared to 61.0 for those who did not.

#### 3.4.2 Individual socialization

**Figure 6:**
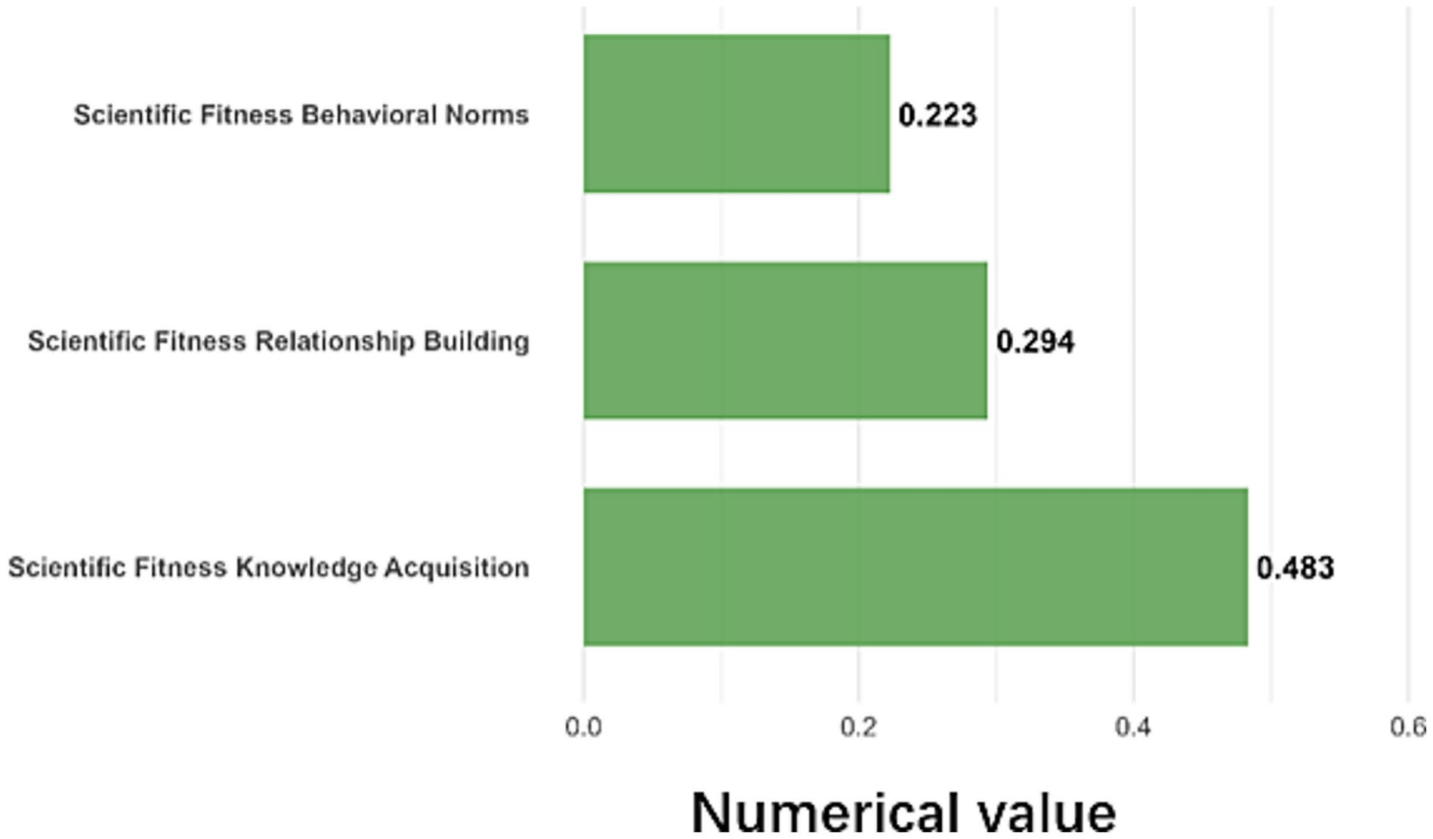
Details of the contribution of the individual socialization.

Individual socialization affected SFL through knowledge acquisition, relationship building, and adherence to behavioral norms (Figure 6). The effect sizes were: knowledge acquisition (*β* = 0.483), relationship building (*β* = 0.294), and behavioral norms (*β* = 0.223). Access to knowledge gad the largest impact, with a 16.6-point difference in the SFL index between the lowest and the highest scoring groups. Using exercise for interpersonal communication and maintaining exercise routines were associated with SFL scores 12.3 and 9.9 points higher, respectively.

#### 3.4.3 Family environment

**Figure 7:**
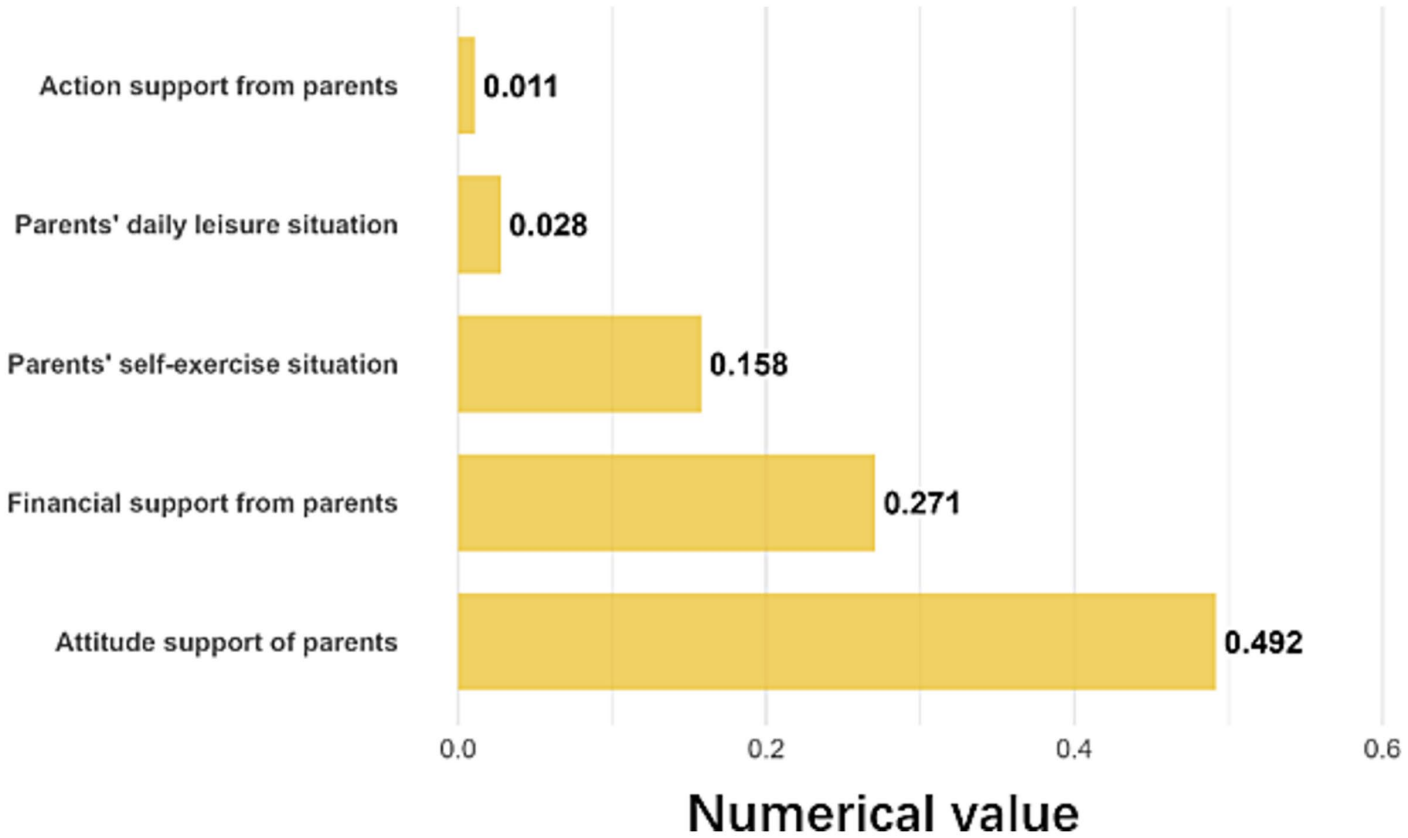
Details of the contribution of the family environment.

Parental attitude and financial support were the most influential components of the family environment (Figure 7). Parental attitude support had the largest effect (*β* = 0.492), followed by financial support (*β* = 0.271). Children with supportive parents had an SFL index 17.5 points higher than those without. Willingness of parents to spend money on physical activity was associated with an SFL index 16.4 points higher.

#### 3.4.4 The school environment

**Figure 8:**
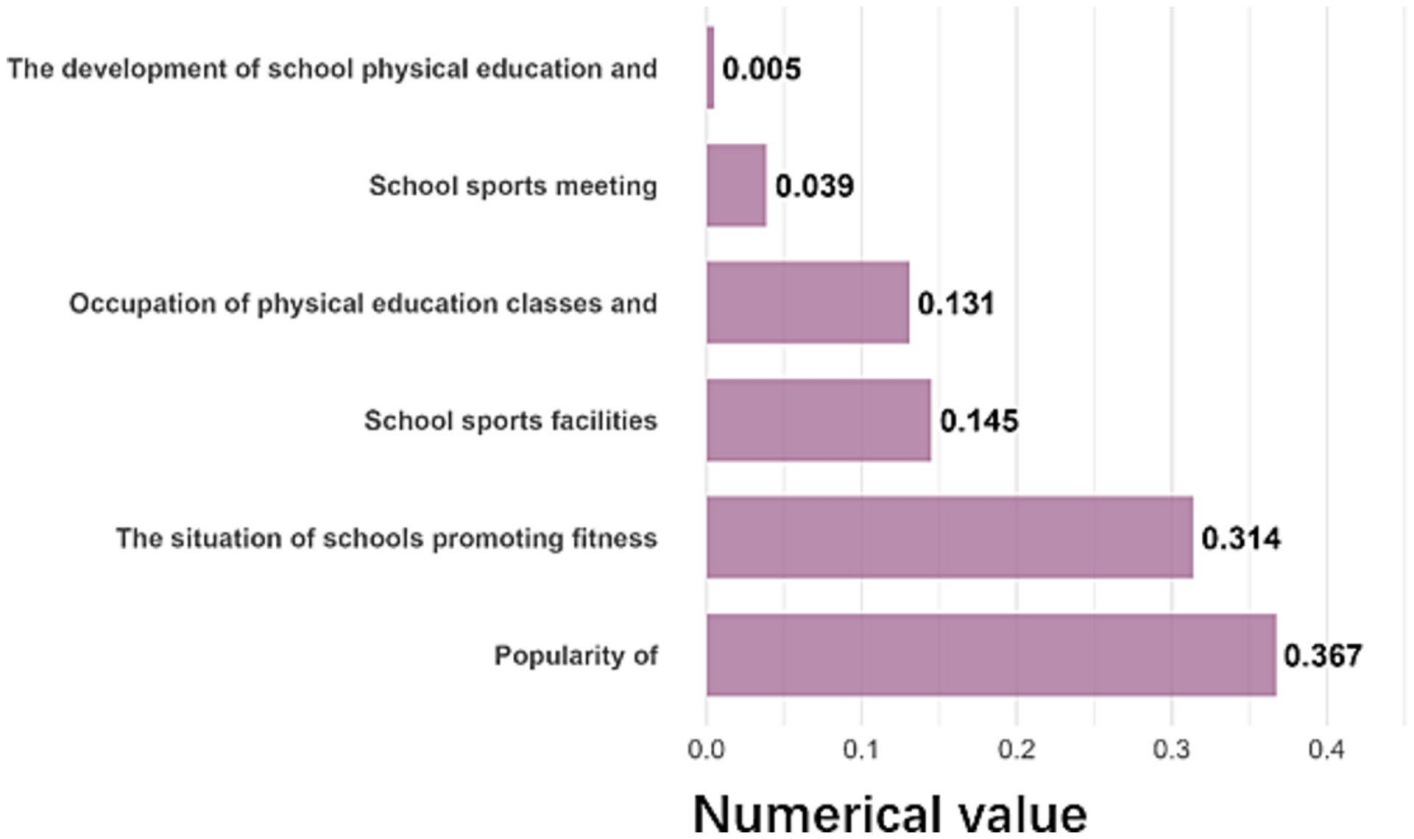
Details of the contribution of the school environment.

The school environment’s influence was most strongly associated with students’ sports popularity and the dissemination of scientific fitness knowledge (Figure 8), with effect sizes of 0.367 and 0.314, respectively. Schools that actively disseminated fitness knowledge had an average student SFL index of 70.7, compared to 59.7 in schools that did not.

#### 3.4.5 The social environment

**Figure 9:**
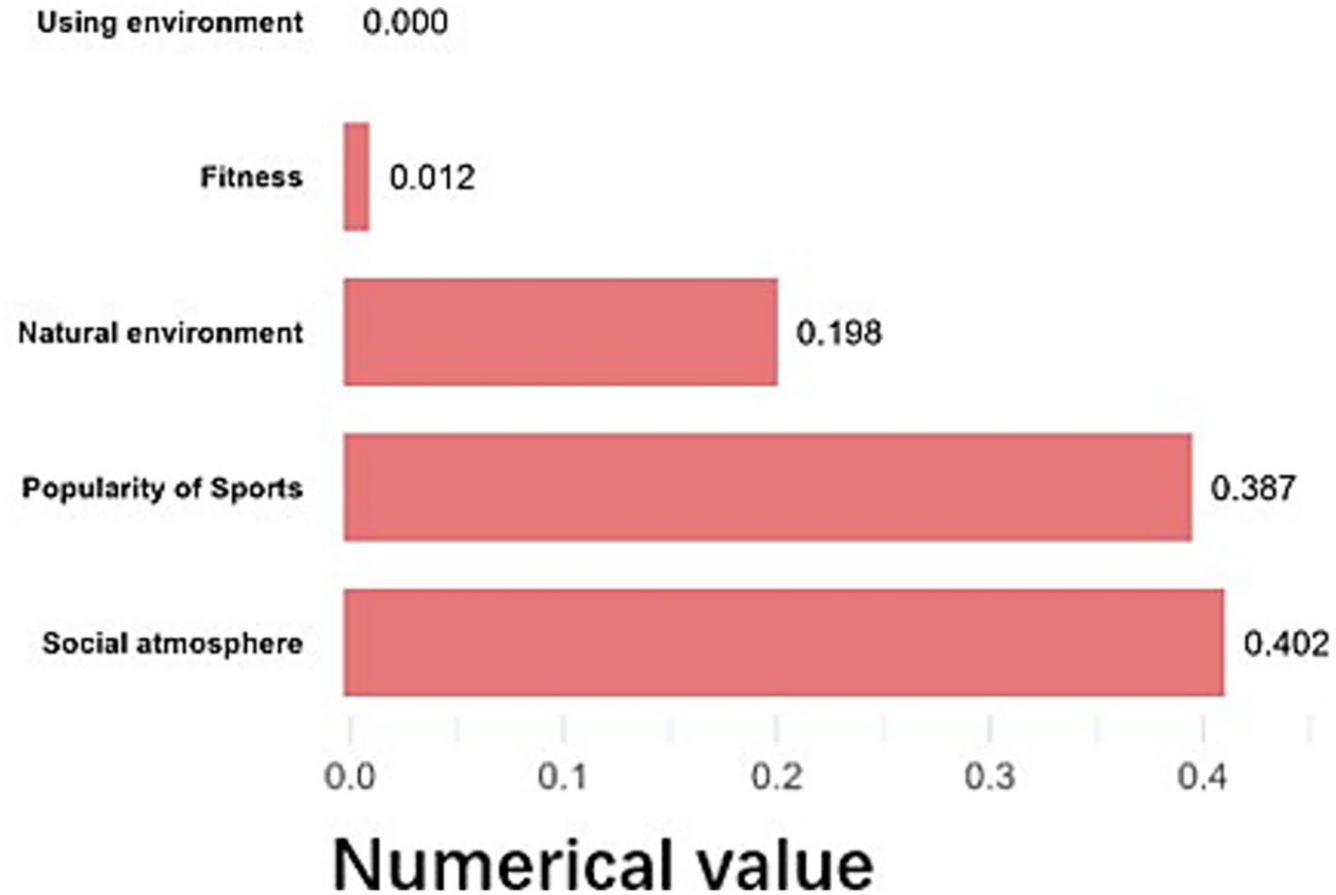
Details of the contribution of the social environment.

The social (community) environment’s influence was primarily driven by the general fitness atmosphere and the presence of fitness organizations (Figure 9), with effect sizes of 0.402 and 0.387, respectively. Children and adolescents who frequently participated in community sports had higher SFL indices.

## 4 Discussion

This study developed and validated an assessment tool for scientific fitness literacy (SFL), a contextualized and operational construct aligned with the international concept of Physical Literacy (PL) but tailored to the specific needs and behaviors of Chinese children and adolescents. Utilizing a large national sample, we evaluated the current status of SFL and examined its key determinants within an integrated social–ecological and self-determination theory (SDT) framework (9).

Our findings reveal that the overall SFL index among Chinese children and adolescents is at a medium-to-low level (65.8/100). Consistent with patterns observed in other contexts (6), attitudes toward fitness were highly positive (82.3), yet this did not translate into commensurate levels of knowledge (59.2) and skills (58.2). This “attitude–behavior gap” (10) suggests that while children recognize the value of fitness, they lack the requisite scientific knowledge and practical skills to engage in safe, effective, and sustained physical activity (11–14). For instance, scores were particularly low in areas such as exercise planning, injury management, and the duration of behavioral habits. This underscores a critical public health implication: interventions must move beyond motivational messaging to include robust, skill-based education and practical guidance that empower youth to translate positive intentions into action (15).

Environmental factors also played significant but distinct roles. The family environment (*β* = 0.211) exerted a substantial influence, primarily through parental attitudes and financial support (Figure 7), corroborating Western studies highlighting the family’s critical role in shaping children’s physical activity patterns (16). In contrast, the school environment showed the lowest predictive utility (*β* = 0.101). While schools ensure a baseline of structural participation (as evidenced by high average scores and a right-skewed distribution), their marginal contribution to enhancing SFL was limited. This may indicate that current school-based physical education, while necessary, may be insufficient in quality or focus to significantly elevate scientific literacy and intrinsic motivation. The social environment (*β* = 0.153), including community atmosphere and organizations, also contributed meaningfully.

This hierarchy of influence—from proximal individual processes to more distal environmental systems—is further illustrated in the mean distribution analyses (Figures 3, 4). The smooth, increasing curves for lifestyle and individual socialization contrast with the “sawtooth” patterns for family, school, and community factors. This visual evidence, supported by the pairwise comparison effect sizes (Tables 5–9; where differences in individual factors often showed large effect sizes Cohen’s d > 0.8), suggests that the impact of environmental factors is more indirect, variable, and potentially mediated by individual perceptions and psychological need satisfaction (as postulated in our framework, Figure 1).

Our findings were consistent with those of previous studies on the importance of PL. A study of adolescents in Hong Kong found that the relationship between perceived PL and physical activity levels was non-significant but was closely related to physical activity negligence when the model was adjusted for individual factors such as gender, grade level, school band, and socioeconomic status (6). Also, a path analysis performed in a Hong Kong study showed that the perceived expectations of the PL of student-athletes were significantly different from those of their coaches. Leadership and relationship were partially predicted and partially mediated by their perceptions of coaching (9).

Other studies from Western countries indicated that the family environment can play an important role (16). For example, only 6% of all children participate in daily physical education classes and only six states offer K-12 physical education in the United States. Family and community engagement can provide opportunities for the children to increase their physical activity participation, which can lead to increased PL among the children (14, 17).

Our results carry important implications for practice and policy. First, the low scores in knowledge and skills call for a curriculum shift in school and community programs toward explicit teaching of exercise science, safety, and self-management skills. Second, the paramount importance of lifestyle and socialization suggests that interventions should foster intrinsic integration of activity into daily life and leverage peer and family networks for modeling and support. Third, the relatively lower marginal utility of school-based factors, coupled with strong parental influence, argues for a strategic reallocation of resources. Investments might yield higher returns if directed toward family-focused interventions (e.g., educating parents and family activity programs) and community capacity building (e.g., accessible sports clubs, and public campaigns), rather than solely increasing school-based mandatory activity time. Our data showing low rates of parental exercise (23.3%) and high rates of foregone activity due to academic pressure (48.6%) pinpoint specific leverage points for such efforts.

### 4.1 Limitations

This study has several limitations that should be acknowledged. First, the cross-sectional design precludes establishing causal relationships among the examined variables. Second, key potential confounding factors—such as socioeconomic status, gender, and objectively measured weight status were not controlled for in the analysis, which may have influenced the observed associations (18). Third, the assessment of scientific fitness literacy (SFL) relied solely on self-reported measures, which are susceptible to biases such as social desirability and recall errors. Future studies would benefit from incorporating objective assessments of SFL (e.g., motor competence tests, accelerometer-based activity monitoring) to better understand the underlying mechanisms. Additionally, longitudinal or intervention-based designs are needed to clarify the directional and causal links between SFL and its associated factors. Despite these limitations, the strengths of this study include its large, nationally representative sample, which enhances the generalizability of the findings and provides a valuable overview of SFL across the population.

### 4.2 Implications

Our study has important implications. It provides a good starting point and an *a priori* hypothesis for further studies. In addition, it provides policymakers with solid evidence to develop related policies and strategies for children’s physical activity in China.

## 5 Conclusion

This study developed a novel assessment model for scientific fitness literacy (SFL) and applied it to a national sample of Chinese children and adolescents. The findings indicate that the current level of SFL is suboptimal, characterized by a significant disparity between positive attitudes and deficient knowledge/skills. An integrated analysis revealed that individual factors (lifestyle and socialization) are the most potent predictors of SFL. In contrast, environmental factors show a gradient of influence from family to society to school. The school environment, despite high levels of structural participation, demonstrates the lowest marginal efficacy in enhancing SFL quality.

These results challenge an over-reliance on school-centric strategies and highlight the urgent need for a more balanced approach. Enhancing SFL requires synchronized development across cognitive, attitudinal, skill-based, and behavioral domains. Future efforts should prioritize: (1) integrating scientific fitness education into curricula to bridge the knowledge-skills gap; (2) developing family- and community-based programs that support positive role-modeling and provide accessible opportunities; and (3) fostering a cultural shift that values and integrates physical activity into daily life beyond compulsory settings. This study provides a theoretical foundation and empirical basis for academics and policymakers to develop targeted, effective guidelines and interventions to advance the scientific fitness literacy of Chinese youth.

## Data Availability

The original contributions presented in the study are included in the article/supplementary material, further inquiries can be directed to the corresponding author.
